# Health Risk Assessment of Ambient PM_2.5_-Bound Metals During Haze Seasons in Urban and Rural Chiang Mai, Thailand

**DOI:** 10.3390/toxics14100869

**Published:** 2026-09-30

**Authors:** Kawinwut Somsunun, Teetawat Santijitpakdee, Turenjai Doolgindachbaporn, Vanisa Surapipith, Sawaeng Kawichai, Tippawan Prapamontol

**Affiliations:** 1 School of Health Sciences Research (SHSR), Research Institute for Health Sciences (RIHES), Chiang Mai University, Chiang Mai 50200, Thailand; kawinwut.som@cmu.ac.th (K.S.); santijitpakdee.t@gmail.com (T.S.); 2 Office of Research Administration, Chiang Mai University, Chiang Mai 50200, Thailand; 3 Department of Environmental Science, Faculty of Science, Khon Kaen University, Khon Kaen 40002, Thailand; turdoo@gmail.com; 4 Regional Resource Centre for Asia and the Pacific at Asian Institute of Technology (AIT RRC.AP), Pathum Thani 12120, Thailand; vanisa.surapipith@rrcap.ait.ac.th

**Keywords:** PM_2.5_-bound metals, haze season, health risk assessment, source identification, urban and rural

## Abstract

Seasonal haze in northern Thailand raises concerns about the health impacts of PM_2.5_-bound metals. This study assessed PM_2.5_-bound metals and inhalation health risks across three contrasting land use environments during the haze season in Chiang Mai Province, Thailand: Chiang Mai University (CMU; an urban site), Mae Chaem (MC; an agricultural rural site), and Chom Thong (CT; a roadside rural site). In total, 101 24 h PM_2.5_ samples were collected during the haze season from 15 January to 22 April 2020. CT showed the highest median PM_2.5_ concentrations (64.9 µg m^−3^), metal enrichment, and overall estimated health risks. Notably, although MC (42.1 µg m^−3^) had lower PM_2.5_ than CMU (50.3 µg m^−3^), its non-carcinogenic hazard index (HI = 2.10) and cancer risk (TCR = 2.37 × 10^−4^) exceeded those of CMU (HI = 1.34; TCR = 1.18 × 10^−4^), indicating that PM_2.5_ mass alone may not adequately capture differences in estimated health risks. HI exceeded the reference level (HI > 1) at all sites, with the highest HI at CT (2.69), and TCR exceeded the benchmark (1 × 10^−4^) at all sites, with the highest TCR at CT (3.63 × 10^−4^) and chromium as the dominant contributor. Se, Cd, and As showed the strongest anthropogenic enrichment, and PCA identified potential source-related associations consistent with biomass burning, traffic emissions, agricultural activities, and crustal or road dust. These findings show that agricultural and roadside rural environments may present distinct health risk profiles that are not captured by PM_2.5_ mass alone, underscoring the need for composition-specific mitigation and public health interventions across the urban–rural continuum in northern Thailand.

## 1. Introduction

Fine particulate matter (PM_2.5_) is one of the most critical environmental health issues worldwide, penetrating deep into the respiratory tract and contributing to cardiovascular disease, respiratory disease, and premature mortality [1,2,3]. Beyond mass concentration, PM_2.5_-bound toxic trace metals—including arsenic (As), cadmium (Cd), chromium (Cr), nickel (Ni), and lead (Pb)—are persistent and bioaccumulative and can act through oxidative stress and genotoxic pathways, increasing risks of lung cancer and chronic disease, even at low ambient concentrations, particularly among children, the elderly, and vulnerable populations [4,5,6,7,8].

Northern Thailand, and Chiang Mai Province in particular, experiences recurrent and severe haze episodes almost every dry season, typically from January to April. These haze events are driven by a combination of local and transboundary biomass burning, agricultural residue burning, forest fires, traffic, and other anthropogenic sources [9,10,11]. As a result, PM_2.5_ concentrations during the haze season frequently exceed national and World Health Organization (WHO) air quality guidelines, prompting public health warnings and temporary control measures [12,13]. Previous studies in northern Thailand have demonstrated strong associations between elevated particulate levels and increased morbidity, mortality, and burden of disease. However, most of these investigations have focused on PM mass and conventional air pollutants rather than on the specific contribution of PM_2.5_-bound metals.

Within Chiang Mai Province, population exposure patterns and emission profiles differ substantially between urban and rural environments. Urban areas, such as the city center around Chiang Mai University, are influenced by dense traffic, urban combustion sources, and local biomass burning [14], whereas rural districts are more strongly affected by open burning of agricultural residues, forest fires, and roadside emissions along major inter-provincial routes [15,16,17]. Comparative urban–rural studies reinforce this pattern. In the North China Plain, urban and rural sites showed diverging trends in PM_2.5_ mass versus metal reduction, with industrial emissions persistently dominating urban risk and rural risk sources shifting dynamically [18]. In Taiyuan, China, Pb and Zn were higher at a rural site than an urban site during a haze episode [19], and a South Korean comparison found significant urban–rural differences in most trace elements [20]. These findings confirm that source composition, not urban–rural classification alone, drives metal-related health risk, yet most studies use a single paired urban–rural site and treat rural sites as homogeneous, limiting insight across the full urban-to-rural continuum.

Importantly, rural environments are not homogeneous. Agricultural districts differ substantially in source profile from roadside rural areas along major transportation corridors, where traffic and industrial activities can be equally or more influential, producing distinct metal compositions and spatially heterogeneous health risks. Three sites were therefore intentionally selected to capture this contrast: CMU (an urban site; traffic and urban combustion), MC (an agricultural rural site; open burning and soil resuspension), and CT (a roadside rural site; traffic emissions, road dust resuspension, and nearby mineral-related activity). Comparing these three environments provides an opportunity to examine how differences in local land use and emission characteristics modify the metal composition and potential health risks of haze-associated PM_2.5_. Nevertheless, there is still limited quantitative information on PM_2.5_-bound metal levels and associated inhalation health risks during haze episodes in Chiang Mai’s contrasting urban and rural micro-environments.

Robust health risk assessment of PM_2.5_-bound metals requires measurement of metal concentrations, evaluation of contamination relative to background concentrations, identification of major sources, and application of standard risk models. Indices such as EF and Igeo, together with PCA, can help characterize geochemical enrichment and potential source-related associations, while exposure assessment models estimate non-carcinogenic and carcinogenic risks from inhalation. However, most previous studies have examined these dimensions individually or compared urban versus rural settings using a single paired-site design, rarely integrating PM_2.5_ mass, metal composition, contamination indices, source signals, and health risk across different land use contexts within one haze episode. Chiang Mai, with its contrasting urban, agricultural–rural, and roadside–rural environments, provides an important case study to address this gap. Therefore, this study aimed to quantify PM_2.5_ and PM_2.5_-bound metals, assess contamination and source profiles, and estimate inhalation health risks across these three sites during the haze season, offering an integrated, spatially resolved evidence base to inform source-specific haze mitigation policy in northern Thailand.

## 2. Materials and Methods

### 2.1. Study Area and Sampling Sites

**Figure 1:**
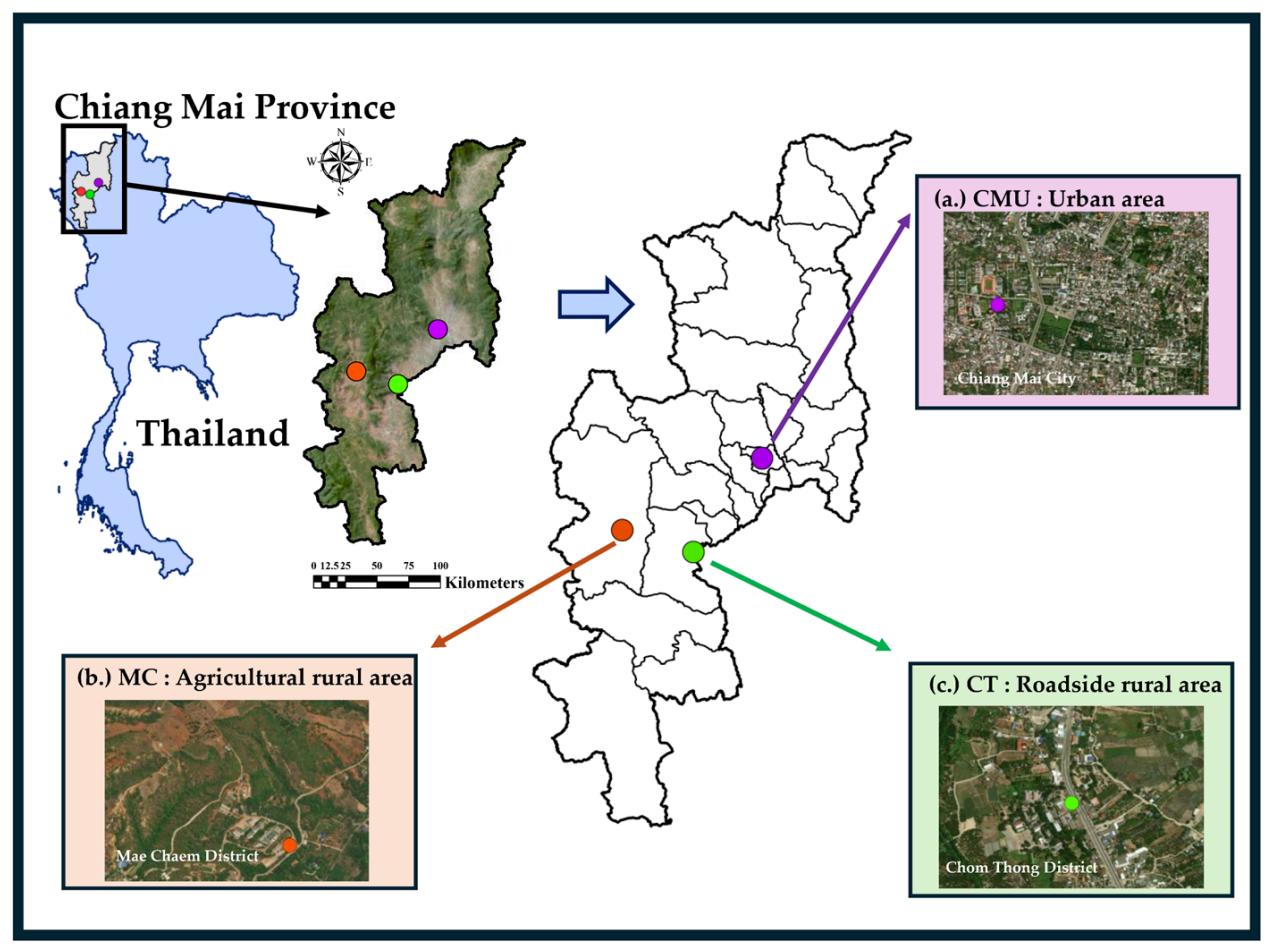
Study area and sampling sites: (**a**) CMU, an urban/university area, (**b**) MC, an agricultural rural area, and (**c**) CT, a roadside rural area.

Ambient PM_2.5_ was sampled in Chiang Mai Province, northern Thailand, which experiences recurrent air pollution during the dry season each year [9,11,21]. Sampling was conducted at three fixed monitoring sites representing distinct land use characteristics (Figure 1): (1) Chiang Mai University (CMU), located in Mueang District, representing an urban mixed residential–educational environment (18°47.71602′ N, 98°57.450258′ E; 332 m MSL); (2) Mae Chaem District (MC), representing an agricultural rural site influenced by open burning and crop-related activities (18°29.86938′ N, 98°22.79406′ E; 530 m MSL); and (3) Chom Thong District (CT), representing a roadside rural site located adjacent to a mining road and characterized by substantial traffic emissions along a major transportation corridor (18°24.4194′ N, 98°40.45746′ E; 301 m MSL). Sampling was conducted from 15 January to 22 April 2020, covering the dry-season haze period in northern Thailand, a period characterized by recurrent biomass burning and elevated hotspot activity throughout the region [22].

### 2.2. PM_2.5_ Sampling

A total of 101 PM_2.5_ samples were collected using mini-volume air samplers (AirMetrics, TAS-5.0, Eugene, OR, USA) operating at a flow rate of 5 L min^−1^. The samplers were equipped with polytetrafluoroethylene (PTFE) filters (Whatman PM_2.5_ PTFE with PP support ring, 46.2 mm diameter, 2.0 μm pore size, Cytiva, Marlborough, MA, USA). Sampling was conducted over 24 h integrated periods to determine the daily average concentrations at each site during the haze season. The filters were pre-conditioned and weighed before and after sampling under controlled temperature and humidity conditions to determine PM_2.5_ mass gravimetrically. Post-sampling, the filters were placed in individual Petri slide dishes, sealed in zip-lock bags, and stored in a refrigerator at 4 °C prior to chemical analysis. Field blanks were collected throughout the campaign to account for potential contamination and handling artifacts.

### 2.3. PM_2.5_-Bound Metal Analysis

The following 13 metals were quantified in the PM_2.5_ samples: aluminum (Al), arsenic (As), barium (Ba), cadmium (Cd), chromium (Cr), copper (Cu), iron (Fe), potassium (K), manganese (Mn), nickel (Ni), lead (Pb), selenium (Se), and zinc (Zn). These target analytes encompass both toxic trace metals (As, Cd, Cr, Ni, Pb, and Se) and major or crustal metals (Al, Ba, Fe, K, Mn, Zn, and Cu).

PM_2.5_-bound metal analysis was conducted following previous studies [23,24]. Briefly, each PTFE filter sample was placed into a digestion vessel and digested with 9 mL of concentrated nitric acid (70% HNO_3_, RCI Labscan, Bangkok, Thailand) and 1 mL of concentrated hydrogen peroxide (30% H_2_O_2_, Merck, Darmstadt, Germany) using a microwave digestion system (ETHOS UP, SK-15 rotor, Milestone Inc., Sorisole, Italy) operated at 190 °C for 35 min. After cooling to room temperature, the digests were quantitatively transferred to volumetric flasks, diluted to 25 mL with Milli-Q water (18.2 MΩ·cm), and filtered through 0.2 μm nylon membrane filters (Agilent Technologies, Santa Clara, CA, USA) to remove insoluble particulates.

The metal concentrations were determined in triplicate using inductively coupled plasma optical emission spectroscopy (ICP-OES; Agilent 5800, Santa Clara, CA, USA). Calibration curves were established using certified multi-element standard solutions (Agilent Technologies, Santa Clara, CA, USA), exhibiting excellent linearity (r^2^ > 0.999) across the analytical range. Method accuracy was verified using urban particulate matter standard reference material (NIST SRM 1648a, Gaithersburg, MD, USA); the recoveries for the target analytes ranged from 60.8% to 88.6%. The quality control (QC) of metal analysis is shown in Table S1. Procedural blanks, duplicate digestions, and replicate instrumental measurements were routinely performed to ensure analytical quality control and precision.

### 2.4. Contamination Assessment

#### 2.4.1. Enrichment Factor (EF)

To assess the degree of the anthropogenic enrichment of metals in PM_2.5_, enrichment factors (*EFs*) were calculated. For each target element *x*, the *EF* was defined according to Equation (1):

(1) $$\mathrm{EF}=\frac{{\left(\frac{C_{x}}{C_{\mathrm{ref}}}\right)}_{{\mathrm{PM}}_{2.5}}}{{\left(\frac{B_{x}}{B_{\mathrm{ref}}}\right)}_{\mathrm{background}}}$$

where *C_x_* and *C_ref_* are the concentrations of the target element and the reference element in the PM_2.5_ samples, respectively, while *B_x_* and *B_ref_* represent their corresponding background concentrations in the upper continental crust [25]. Aluminum (Al) was selected as the reference crustal element due to its high abundance in the crust and minimal anthropogenic contribution. Background concentrations were obtained from two sources: for 11 metals (Al, As, Ba, Cr, Cu, Fe, K, Mn, Ni, Pb, and Zn), local background values were derived from highland coffee soils in Chiang Mai [26]; for Cd and Se, which were unavailable in that dataset, upper continental crust values from Taylor and McLennan were used instead [27,28]. The background concentration parameters for each element are shown in Table S2. The calculated *EF* values were categorized into five enrichment levels: *EF* < 1 (definitively unenriched, reflecting natural or crustal sources), 1 ≤ *EF* < 2 (slight anthropogenic influence), 2 ≤ *EF* < 10 (moderate enrichment), 10 ≤ *EF* < 100 (significant enrichment from anthropogenic activities), and *EF* ≥ 100 (extremely high enrichment, indicating severe impact from human activities). In this study, the term anthropogenic enrichment refers to *EF* values exceeding 10 (*EF* > 10), indicating that a metal is predominantly influenced by anthropogenic rather than crustal sources [7].

#### 2.4.2. Geoaccumulation Index (Igeo)

The geoaccumulation index (*Igeo*) was applied to further characterize the degree of metal accumulation in PM_2.5_ relative to crustal background levels [29,30]. The *Igeo* for each target metal *x* was computed using Equation (2):

(2) $$I_{\mathrm{geo},i}=\log_{2}\left(\frac{C_{x}}{1.5\times B_{x}}\right)$$

where *C_x_* is the measured concentration of element *x* in the PM_2.5_ samples, and *B_x_* represents its corresponding background concentration in the upper continental crust [27,28]. A constant factor of 1.5 was introduced to account for potential natural variations in the crustal background matrix. The calculated *Igeo* values were interpreted using standard contamination classes [31].

### 2.5. Potential Source Identification

Principal component analysis (PCA) with varimax rotation was performed on the standardized metal concentration dataset to qualitatively identify potential sources of PM_2.5_-bound metals. Prior to PCA, the variables were examined for normality and, where necessary, log-transformed to reduce skewness and satisfy statistical assumptions. Components with eigenvalues greater than 1.0 were retained. Source interpretations were based on combinations of metals with strong factor loadings on the rotated component matrix (≥0.60) rather than on individual elements alone. Source categories were assigned based on established tracer roles reported in the literature (Table S3). When a component exhibited high loadings for tracers of more than one source category, it was interpreted as reflecting overlapping or mixed sources rather than a single dominant source.

### 2.6. Statistical Analysis

Descriptive statistics, including the mean, median, standard deviation, and range, were calculated for PM_2.5_ mass and metal concentrations at each sampling site. All PM_2.5_ comparisons refer to the WHO (2021) 24 h mean guideline value of 15 µg m^−3^ [12]. Differences in concentrations and contamination indices among sites and sampling periods were evaluated using either one-way analysis of variance (ANOVA) or its non-parametric equivalent (e.g., the Kruskal–Wallis test), depending on data normality and homogeneity of variance. Pearson or Spearman correlation analysis was conducted to explore the relationships among metals and between individual metals and PM_2.5_ mass. All statistical analyses were performed using standard statistical software, with statistical significance defined as *p*-value < 0.05.

### 2.7. Health Risk Assessment of PM_2.5_-Bound Metals

The inhalation exposure concentration (*EC_inh_*) was first estimated for each PM_2.5_-bound element for the adult population using the measured concentrations and the corresponding exposure parameters. The resulting *EC_inh_* values were then used to estimate non-carcinogenic risk, expressed as the hazard quotient (*HQ*) and hazard index (*HI*), and carcinogenic risk, expressed as the individual inhalation cancer risk (*CR_inh_*) and total cancer risk (*TCR*). The inhalation exposure concentration was calculated using Equation (3):

(3) $${\mathrm{EC}}_{\mathrm{inh}}=\frac{C_{x}\;\times \;E_{i}\times \mathrm{ET}\times \mathrm{EF}\times \mathrm{ED}}{{\mathrm{AT}}_{n}}$$

where *EC_inh_* is the inhalation exposure concentration; *C_x_* is the concentration of each metal *x* in PM_2.5_ (µg m^−3^); *Ei* is the fraction of PM_2.5_ deposition absorbed in the lungs (*Ei* = 0.48); *ET* is the exposure time (24 h day^−1^); *EF* is the exposure frequency (*EF* = 350 d year^−1^); *ED* is the exposure duration (*ED* = 24 years); and *AT_n_* is the averaging time, calculated as *ED* × 365 d year^−1^ × 24 h day^−1^ (*AT_n_* = 210,240 h) for non-carcinogenic risk and 70 years × 365 d year day^−1^ × 24 h day^−1^ (*AT_n_* = 613,200 h) for carcinogenic risk [32,33]. The health risk assessment targeted the general adult resident population (body weight, *BW* = 70 kg), using standard USEPA default exposure parameters. A full parameter summary, including metal-specific reference concentrations (*RfC*) and inhalation unit risk (*IUR*) factors, is provided in Tables S4 and S5.

#### 2.7.1. Non-Carcinogenic Risk Assessment of Heavy Metals in PM_2.5_

The non-carcinogenic health risk of PM_2.5_-bound heavy metals was evaluated using the hazard quotient (*HQ*). The *HQ* is defined as the ratio between the estimated exposure concentration of a tract metal *x* in PM_2.5_ over a specified period and its corresponding reference concentration (*RfC_x_*) for the same exposure duration [32,34,35]. This approach is used to assess potential adverse health effects other than cancer that may result from long-term exposure.

The *HQ* for each metal was calculated using Equation (4) by dividing the inhalation exposure concentration (*EC_inh_*) by the inhalation reference concentration (*RfC*):

(4) $$HQ_{x}=\frac{EC_{x}}{\mathrm{Rf}C_{x}}\times 1000\;\mu g/\mathrm{mg}$$

The hazard index (*HI*) was used to estimate the combined non-carcinogenic risk from simultaneous exposure to multiple metals. The *HI* is the sum of the *HQ* values for all metals in the mixture, as shown in Equation (5):

(5) $$\mathrm{HI}=\sum_{x}\mathrm{HQ}$$

The *RfC* values (mg m^−3^) for each metal were obtained from the 2023 Regional Screening Level (*RSL*) Summary Table [33,34], as listed in Table S5. *HQ* or *HI* values greater than 1.0 indicate a potential for chronic health effects, whereas values below 1.0 suggest that significant non-carcinogenic effects are unlikely.

#### 2.7.2. Carcinogenic Risk Assessment of Tract Metals in PM_2.5_

Carcinogenic risk associated with inhalation exposure to carcinogenic metals (As, Cd, Cr, Ni, and Pb, where inhalation unit risks are available) was estimated using exposure concentration and inhalation unit risk (*IUR*) factors, consistent with the ATSDR and USEPA approaches [32,36]. This metric represents the probability of developing cancer due to long-term exposure to carcinogenic metals and was calculated using Equation (6):

*CR_inh_* = *EC_inh_* × *IUR_x_* (6)

where *CR_inh_* is the excess lifetime cancer risk of metal *x,* and *IUR_x_* is the inhalation unit risk ((µg m^−3^)^−1^) for metal *x*, obtained from the 2023 RSL Summary Table [34], as shown in Table S5.

The total cancer risk (*TCR*) was calculated by summing the individual carcinogenic risks of the target metals in PM_2.5_, including As, Cd, Cr, Ni, and Pb, as shown in Equation (7) [34,37]:

(7) $$\mathrm{TCR}=\sum_{x}\mathrm{CR}$$

A *CR_inh_* and *TCR* value below 1 × 10^−6^ was considered negligible, values between 1 × 10^−6^ and 1 × 10^−4^ were considered within the acceptable or tolerable risk range, and values exceeding 1 × 10^−4^ were considered indicative of an unacceptable lifetime carcinogenic risk [37,38].

## 3. Results and Discussion

### 3.1. PM_2.5_ and Metal Concentrations

Table 1 summarizes the overall descriptive statistics of PM_2.5_ and PM_2.5_-bound metal concentrations for all samples collected in Chiang Mai Province. Ambient PM_2.5_ samples collected during the haze season (15 January–22 April 2020) in Chiang Mai Province showed an overall mean concentration of 60.65 µg m^−3^, with a median of 52.43 µg m^−3^ and a maximum of 295.99 µg m^−3^, indicating substantial temporal variability and frequent severe haze episodes. Of the 101 24 h PM_2.5_ samples, 90 (89%) exceeded the WHO (2021) 24 h Air Quality Guideline level (15 µg m^−3^), and 67 (66%) exceeded Thailand’s current 24 h National Ambient Air Quality Standard (37.5 µg m^−3^) [12,39]. Exceedance rates for the WHO guideline were highest at CT (95%), followed by MC (83%) and CMU (88%), while exceedance rates for the Thai standard followed a similar spatial pattern (CT 80%, MC 58%, and CMU 54%).

Among the measured PM_2.5_-bound metals, Al, K, and Fe were the most abundant, with mean concentrations of 2948.35, 418.98, and 335.58 ng m^−3^, respectively, followed by Zn, Cr, Se, As, Ba, Mn, Pb, Ni, Cu, and Cd. Although toxic trace metals, including As, Cd, Cr, Ni, Pb, and Se, were detected at comparatively lower concentrations, they remain of concern because of their well-established toxicological effects.

**Figure 2:**
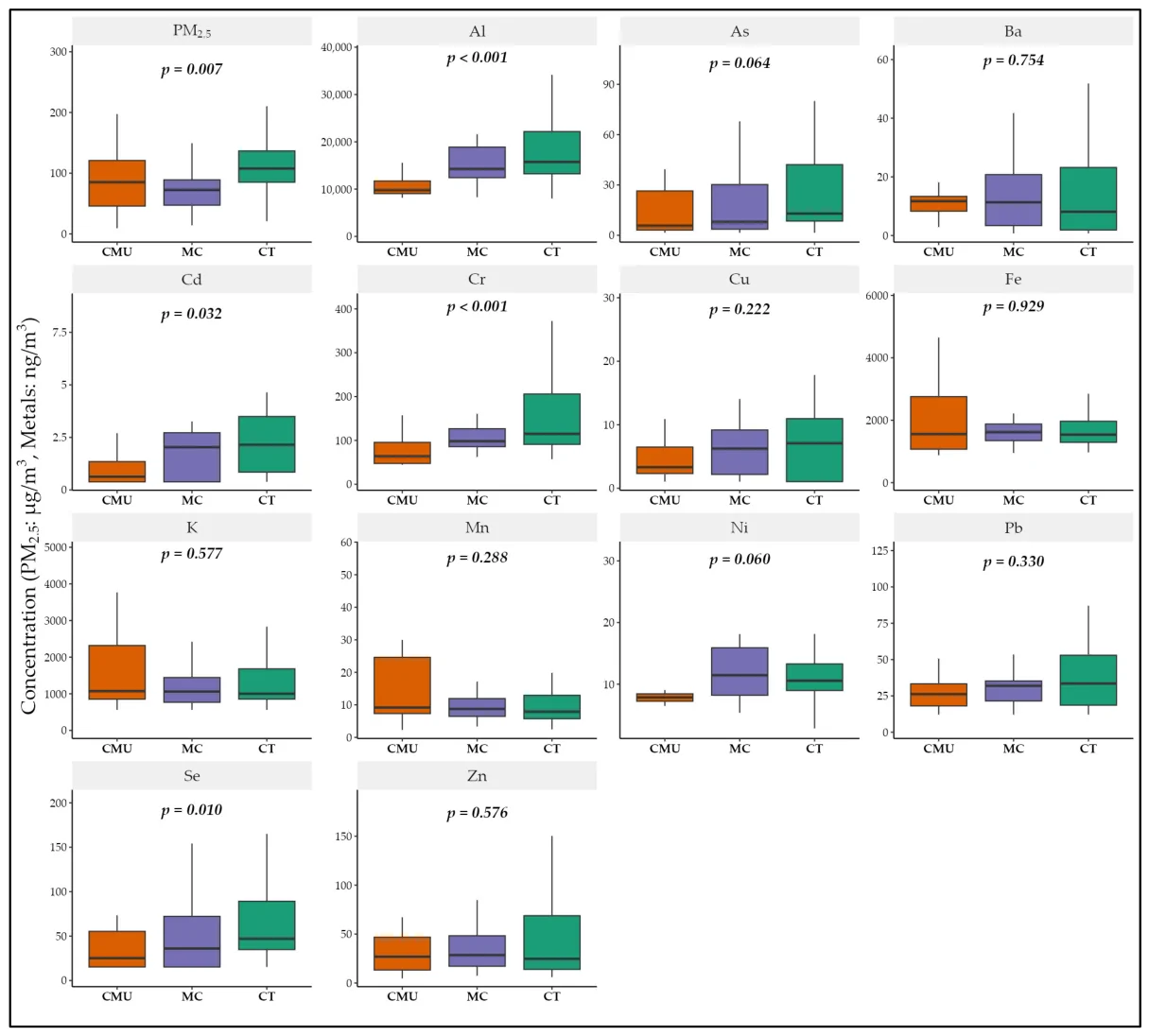
Comparisons of PM_2.5_ and PM_2.5_-bound metal concentrations in three different land use areas in Chiang Mai Province (15 January to 22 April 2020).

Across the three sampling sites, PM_2.5_ concentrations differed significantly (*p* = 0.007), with the highest median concentration observed at CT (64.90 µg m^−3^), followed by CMU (50.35 µg m^−3^) and MC (42.10 µg m^−3^) (Figure 2 and Table S6). This pattern indicated that the roadside rural site experienced the greatest PM_2.5_ burden during the haze season, likely reflecting the combined influence of regional biomass burning and local traffic-related emissions. Significant spatial differences were also observed for Al, Cd, Cr, and Se. CT consistently exhibited the highest median concentrations of PM_2.5_, Al, Cr, and Se, whereas Cd concentrations were higher at both MC and CT than at CMU. Pairwise comparisons further revealed that PM_2.5_ concentrations differed significantly only between CT and MC (*p*-value = 0.001) (Table S7), indicating that local geographical characteristics and emission sources likely contributed to the observed spatial variation. MC is in a mountainous area with a relatively low traffic density, whereas CT is situated along a major transportation corridor connecting southern Chiang Mai with the city. Topographic conditions may also have influenced PM_2.5_ accumulation, as CT is located on the basin floor, where pollutants are more likely to accumulate [40,41], whereas MC is closer to the basin boundary.

Metal analysis further demonstrated that Al and Cr concentrations were significantly higher at MC and CT than at CMU, suggesting greater contributions from soil-derived and resuspended dust in the rural environments [42,43,44,45]. In addition, elevated Al concentrations may also reflect emissions from fuel combustion associated with agricultural activities, which are more prevalent in MC and CT [46]. Compared with CMU, CT also exhibited significantly higher concentrations of As, Cd, Ni, and Se, indicating additional contributions from biomass burning, wood and oil combustion, agricultural activities, and soil or road dust resuspension [19,45,47,48,49,50].

Compared with studies conducted during similar seasons (Table 2), the mean PM_2.5_ concentration in Chiang Mai was higher than that reported in Khon Kaen, Rayong, Pathum Thani, suburban Jinan (China), and industrial areas in Korea [38,45,51,52,53], but lower than that reported in highly polluted locations, including Ahvaz (Iran) and Chengdu (China) [42,54]. Overall, the concentrations of PM_2.5_-bound metals measured in this study were within the ranges reported worldwide. Notably, Al concentrations were generally higher, whereas Pb concentrations were lower than those reported in many previous studies. The relatively high Al concentration may reflect stronger contributions from crustal materials, soil resuspension, and agricultural activities, while the lower Pb concentration may indicate reduced traffic-related emissions compared with more urbanized or industrialized regions [42,45,46]. This interpretation is further supported by Thailand’s nationwide phase-out of leaded gasoline in 1994, which substantially reduced Pb emissions from vehicular sources, although Pb can still originate from biomass burning, brake and tire wear, and resuspended road or soil dust [55,56,57]. Similarly, concentrations of Cd, Cu, Mn, and Pb were lower than those reported for urban and industrial environments, where these metals are commonly associated with industrial activities, vehicular emissions, and other combustion-related sources [42,45,58,59,60,61,62].

The site-specific results further supported this spatial pattern, with CT (roadside rural site) exhibiting the highest PM_2.5_ concentrations and MC (agricultural rural site) the lowest. CT also had the highest concentrations of most measured metals among the three sampling sites, highlighting it as the most affected microenvironment in the present study. The observed metal profiles indicate that differences in local emission sources contributed to the spatial variation across the urban and rural environments. Compared with the two rural sites, CMU, representing the urban university area, showed lower median concentrations of Al, As, Cd, Cr, Ni, and Se (Figure 2 and Table S6), suggesting a relatively smaller contribution from resuspended mineral dust and agricultural-related emissions during the study period [19,42,45,46,48]. In contrast, MC, the agricultural rural site, exhibited higher median concentrations of Al and Cr than CMU, consistent with contributions from biomass burning, soil resuspension, and agricultural activities during the haze season [42,43,45,46,63]. CT displayed the clearest mixed-source profile. In addition to the highest PM_2.5_ concentration, CT recorded the highest median concentrations of Al, Cr, Cu, K, Se, and Zn, together with elevated levels of As, Cd, and Pb. This metal pattern suggests a combined influence of regional biomass-burning smoke, traffic emissions, roadside dust resuspension, agricultural activities, and potentially mining road-related disturbances [42,44,45,46,48,58,59,63].

### 3.2. Enrichment Factor and Anthropogenic Influence

#### 3.2.1. Enrichment Factor

**Figure 3:**
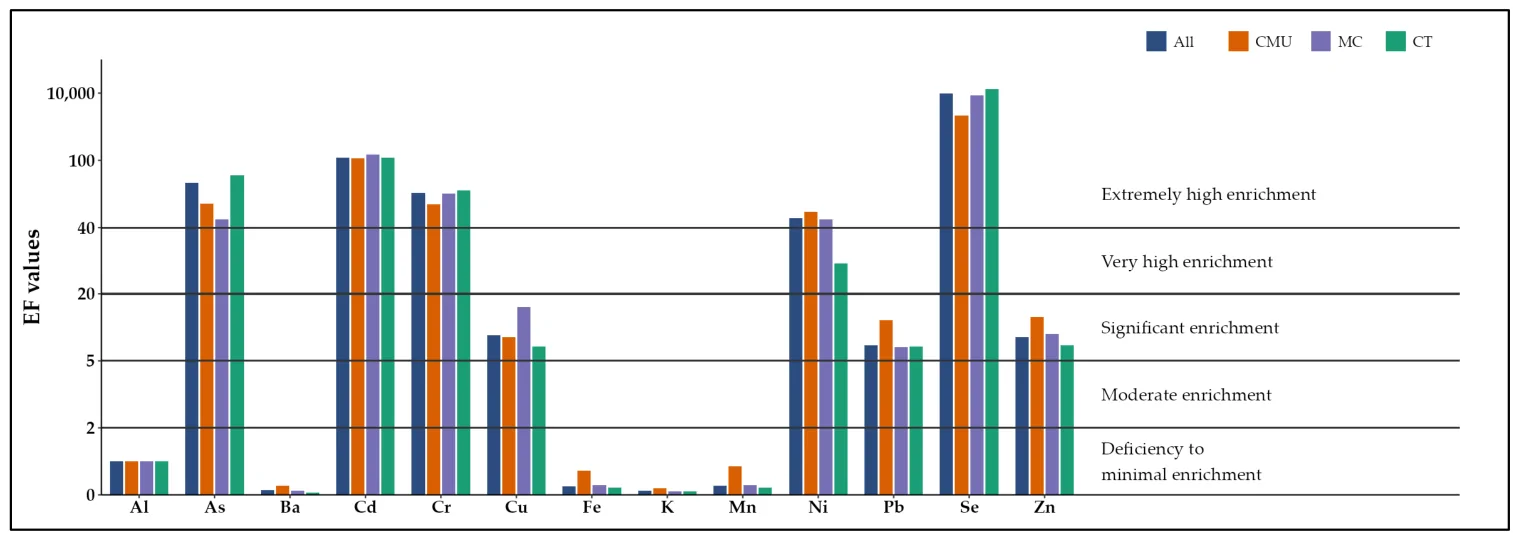
Enrichment factor (EF) values of PM_2.5_-bound metals in Chiang Mai Province.

The enrichment factor (EF) results indicate substantial anthropogenic enrichment for several toxic metals (Figure 3 and Table S8). Extremely high EF values (EF > 100) were observed for Se (EF = 9917.8) and Cd (EF = 528.0), indicating strong anthropogenic contributions. Among the three sampling sites, CT exhibited the highest EF values for Se, whereas the greatest Cd enrichment was observed at MC. Significant enrichment (10 ≤ EF < 100) was also observed for As (EF = 80.06), Cr (EF = 70.67), Ni (EF = 48.31), Cu (EF = 10.69), and Zn (EF = 10.38), indicating considerable anthropogenic influence.

CT is located along a major transportation corridor connecting southern Chiang Mai with the city and lies approximately 27 km from several limestone quarries and stone-crushing facilities [64]. Heavy-duty truck traffic associated with these operations may have contributed to the enrichment of trace metals in PM_2.5_. In addition, mining and mineral-processing activities have been identified as potential sources of atmospheric Se, while limestone injections during combustion processes have been reported to enhance As and Se emissions [65,66]. The exceptionally high Se enrichment observed at CT may also reflect contributions from traffic emissions together with biomass and fossil fuel combustion during the haze season [42,45,47]. Similarly, the elevated As enrichment at CT may be associated with agricultural activities, including agricultural chemical use, as well as road dust resuspension [19,48,58,59]. In contrast, the pronounced Cd enrichment at MC may reflect the influence of wildfire emissions during the study period [49]. The significant enrichment of As, Cr, Cu, and Zn suggested considerable anthropogenic influence. These metals have frequently been linked to traffic-related emissions, fuel combustion, and resuspended road or soil dust, representing plausible sources [19,42,44,45,58,61,67,68]. Pb showed moderate enrichment overall (EF = 8.56; 2 ≤ EF < 10), with the highest EF observed at CMU (EF = 14.13), suggesting a stronger traffic-related influence in the urban area. Although leaded gasoline has been phased out in Thailand for more than three decades, non-exhaust traffic emissions, including brake wear, brake pad abrasion, and engine wear, remain important sources of airborne Pb [55,69,70]. In contrast, Mn, Fe, Ba, and K showed low EF values (EF < 1), indicating predominantly crustal origins with limited anthropogenic influence. These metals are abundant in natural soils and are commonly associated with resuspended mineral dust [44,45,60].

Spatially, the EF values for most highly enriched toxic metals, including As, Cd, Cr, Ni, Pb, Se, and Zn, did not differ significantly among the three sampling sites, indicating that anthropogenic inputs were widespread across the study area during the haze season rather than being restricted to a particular environment. Significant spatial differences (*p* < 0.05) were observed only for Ba, Fe, K, and Mn. However, because these metals are primarily associated with crustal materials or mixed natural and anthropogenic sources, their spatial variability does not alter the overall interpretation that toxic trace metals were substantially enriched relative to the crustal background.

It should also be noted that the highland coffee soil background values [26], while more locally representative of the study region than the global upper continental crust averages, were derived from a mountainous agricultural soil matrix and may not fully capture background variability across the urban (CMU) and lowland roadside (CT) environments included in this study. Background values for Cd and Se were unavailable in that dataset and were instead obtained from global crustal averages [27], introducing an additional source of heterogeneity. Nevertheless, because the enrichment patterns identified for key toxic metals (Se, Cd, As, and Cr) were consistently supported by both EF and Igeo, the overall interpretation of anthropogenic enrichment is unlikely to be materially affected by the choice of background dataset.

#### 3.2.2. Geoaccumulation Index and Contamination Factor

**Figure 4:**
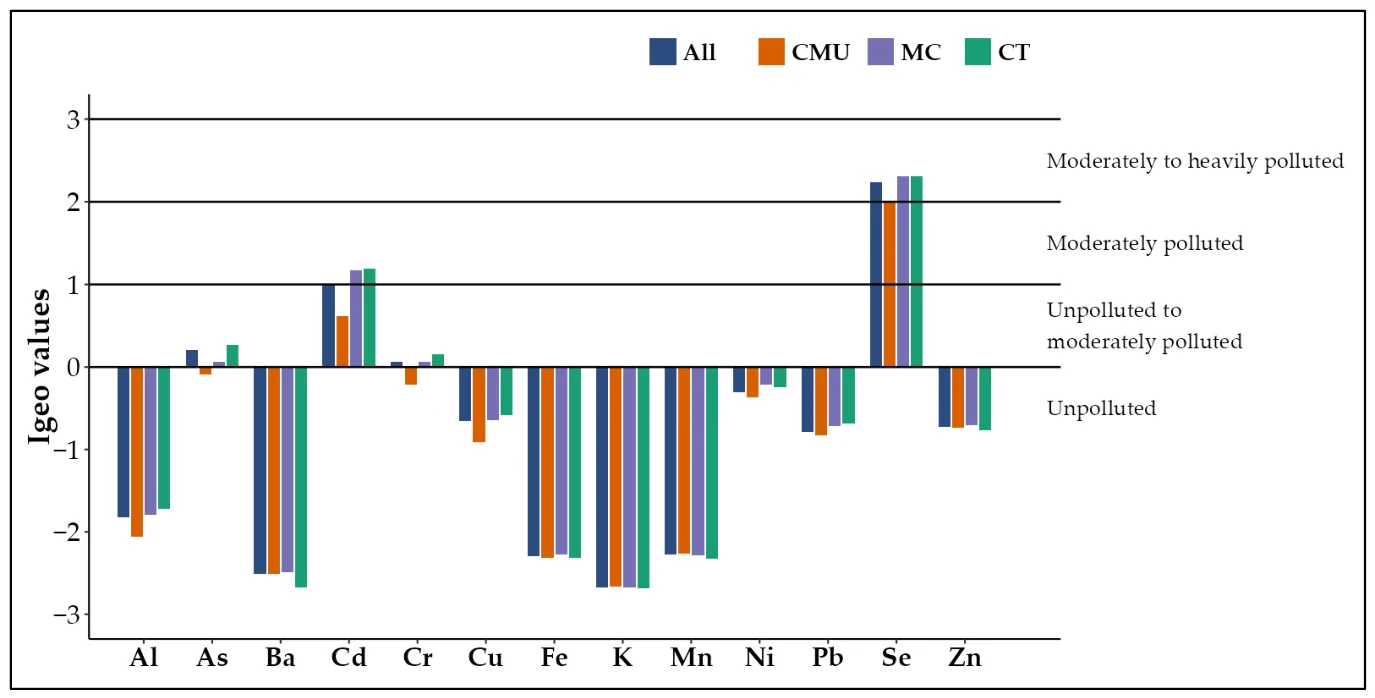
Geoaccumulation index (Igeo) values of PM_2.5_-bound metals in Chiang Mai Province.

The geoaccumulation index (Igeo) results, summarized in Figure 4 and Table S9, were generally consistent with the EF findings but provided a more conservative evaluation of contamination. Positive median Igeo values were identified only for Se (Igeo = 2.24), Cd (Igeo = 1.00), As (Igeo = 0.67), and slightly for Cr (Igeo = 0.06), whereas all other metals generally exhibited negative values (Igeo < 0). According to the Igeo classification, Se was classified as moderately to strongly polluted, Cd as moderately polluted, and As as unpolluted to moderately polluted, while the remaining metals were classified as unpolluted relative to the crustal background.

### 3.3. Potential Source Identification by PCA

To identify the potential sources of PM_2.5_-bound metals, principal component analysis (PCA) was performed. The suitability of the dataset was confirmed by the Kaiser–Meyer–Olkin (KMO) measure of sampling adequacy and Bartlett’s test of sphericity. The overall KMO value was 0.768, indicating moderate-to-good sampling adequacy for factor analysis. Bartlett’s test was statistically significant (χ^2^ = 401.82, degrees of freedom = 78, *p* < 0.001), confirming that the 13 metals were sufficiently correlated to justify PCA. Varimax rotation with Kaiser normalization was applied to facilitate interpretation. Three principal components with eigenvalues greater than 1 were extracted, collectively explaining 77.7% of the total variance (Table 3).

Component 1 accounted for 57.60% of the total variance and was characterized by strong positive loadings for Pb (0.922), K (0.842), Cu (0.751), Zn (0.728), and Mn (0.608), suggesting a predominantly mixed anthropogenic source. The high loading of K is commonly associated with biomass burning and other combustion processes [9,11,71,72,73], whereas Pb, Cu, and Zn are widely recognized as tracers of traffic-related [45,58,60,61,68,69,70] and combustion-related emissions [56,57]. Accordingly, this component was most likely suggestive of the combined influence of haze-related biomass burning and vehicular emissions. This is consistent with regional source apportionment in northern Thailand, where biomass burning (57.9%) and vehicle exhaust (7.8%) were identified as major haze-season contributors, supporting the co-loading of K with Pb, Cu, and Zn as overlapping rather than independent sources [14,56,74,75].

Component 2 explained 10.16% of the total variance and was characterized by high loadings of Cr (0.849), Ba (0.807), Al (0.702), Se (0.675), Cd (0.658), and As (0.644), with a moderate contribution from Fe (0.588). This loading pattern suggested a mixed source influenced by crustal materials, road and soil dust resuspension, traffic-related emissions, agricultural activities, and combustion-derived trace metals. Al, Ba, and Fe are commonly regarded as tracers of mineral and crustal materials [42,43,44,45,63], whereas Cr, Cd, As, and Se are more frequently associated with anthropogenic sources, including traffic-related emissions [38,42,76,77,78], agricultural activities, and combustion processes [45,47,48,49]. Collectively, these loadings suggest that this component was consistent with the combined influence of local dust resuspension and anthropogenic combustion-related inputs. This mixed pattern is plausible given that open burning (agricultural residue and forest fire) accounts for roughly half of annual PM_2.5_ emissions in Chiang Mai Province, and CT’s proximity to limestone quarrying likely adds mining and road dust inputs to this source signature. This is consistent with a previous study that found a high enrichment of Se and Ba at CT [74].

Component 3 explained 9.918% of the total variance and was characterized primarily by high loadings of Ni (0.895) and Mn (0.629), suggesting a more distinct source profile. This component may be associated with biomass and fossil fuel combustion, mechanical abrasion, and site-specific agricultural or mining road activities involving resuspended soil [19,45,47,49,50,57,79]. Studies in northern Thailand found that Ni and Mn may be associated with industrial sources [56]. As with the other components, this grouping reflects statistical co-variation rather than a confirmed source, given the overlapping and seasonally dynamic emissions in this airshed. Overall, the PCA results suggest that PM_2.5_-bound metals in Chiang Mai during the haze season were influenced by multiple overlapping sources, primarily biomass burning, traffic-related emissions, agricultural activities, and crustal or road dust.

### 3.4. Health Risk Assessment

#### 3.4.1. Non-Carcinogenic Health Risk

The non-carcinogenic risk assessment showed that the total hazard index (HI) for inhalation exposure exceeded the safety threshold (HI > 1) at all sampling sites (Table 4). The total HI was 2.14 for all samples combined, with site-specific values of 1.34 at CMU, 2.10 at MC, and 2.69 at CT. These findings indicate a potential non-carcinogenic health risk associated with inhalation exposure to PM_2.5_-bound metals throughout the study area, with the greatest risk occurring at the roadside rural site (CT). Across all sites, Cr, As, Ni, and Al were the primary contributors to the total HI. CT exhibited the highest hazard quotients (HQs) for Cr (8.33 × 10^−1^), As (7.73 × 10^−1^), Ni (4.77 × 10^−1^), and Al (3.76 × 10^−1^), followed by Cd (1.13 × 10^−1^) and Mn (1.02 × 10^−1^). A similar pattern was observed at CMU, although HQ values were consistently lower, with Cr (2.70 × 10^−1^), As (4.22 × 10^−1^), Ni (3.24 × 10^−1^), and Al (1.14 × 10^−1^) contributing most to the total HI of 1.34. The risk profile at MC more closely resembled that at CT than at CMU, particularly for As (5.60 × 10^−1^), Cr (5.44 × 10^−1^), and Ni (5.34 × 10^−1^).

Although MC had a lower PM_2.5_ mass concentration than CMU, its total HI remained above 2 and exceeded that observed at CMU. This finding suggests that inhalation risk was influenced not only by PM_2.5_ mass concentration but also by the site-specific composition of PM_2.5_-bound metals [61,80]. CT exhibited both the highest PM_2.5_ concentration and the highest overall non-carcinogenic risk, indicating that this roadside rural site represented the greatest potential exposure setting among the three sampling locations. These findings further suggest that reducing PM_2.5_ mass alone may not be sufficient to minimize health risks if concentrations of highly toxic trace metals remain elevated during haze episodes.

#### 3.4.2. Carcinogenic Risk

The carcinogenic risk assessment indicated that the total cancer risk (TCR) for all sampling sites combined was 2.59 × 10^−4^ (Table 4). Consistent with the non-carcinogenic risk assessment, the highest TCR was recorded at CT (3.63 × 10^−4^), followed by MC (2.37 × 10^−4^) and CMU (1.18 × 10^−4^). All estimated TCR values exceeded the commonly accepted benchmark of 1 × 10^−4^, indicating a potentially elevated lifetime cancer risk associated with inhalation exposure to PM_2.5_-bound metals during the haze season in Chiang Mai Province.

Chromium (Cr) was the dominant contributor to the total cancer risk (TCR) at all sampling sites. The estimated carcinogenic risk (CR) for Cr was highest at CT (3.60 × 10^−4^), followed by MC (2.35 × 10^−4^) and CMU (1.17 × 10^−4^), with all values exceeding the commonly accepted risk threshold (CR > 1 × 10^−4^) for inhalation exposure. In contrast, Cd contributed CR values within the acceptable or tolerable risk range (1 × 10^−6^ to 1 × 10^−4^), whereas the estimated risks for As, Ni, and Pb were below the negligible risk threshold (CR < 1 × 10^−4^). These results indicate that the estimated carcinogenic risk in the study area was primarily driven by Cr, with the highest contribution observed at CT. Because total chromium was measured analytically, these findings should be interpreted with caution if the risk assessment assumes that all measured Cr is present as the more toxic hexavalent chromium Cr (VI). Under the assumptions applied in the present assessment, Cr remained the predominant contributor to the estimated carcinogenic risk.

The inhalation health risks estimated in this study were generally higher than those reported in previous studies from Japan, China, Malaysia, and Indonesia [45,81,82,83,84] but lower than those reported from Vietnam for non-carcinogenic risk [84,85]. Within Thailand, the estimated non-carcinogenic risks exceeded those reported in previous studies conducted in Upper Northern Thailand (UNT) and Pathum Thani [23,24,52,84] but were comparable to those reported in Rayong and Khon Kaen Provinces [38,51,59]. In contrast, the estimated carcinogenic risk was higher than that reported in Pathum Thani and Rayong Province but lower than that reported in previous studies from UNT, industrial areas of Rayong Province, and Khon Kaen Province [23,24,38,51,52,59,84].

These findings indicate that haze-season exposure to PM_2.5_-bound elements in Chiang Mai is not uniformly distributed across different land use settings. CT, the roadside rural site, consistently exhibited the highest PM_2.5_ concentrations, elevated levels of several toxic elements, the highest HI, and the highest TCR, suggesting that populations living in roadside rural environments may experience greater inhalation exposure than those in the urban university setting during severe haze episodes. MC also exhibited elevated contamination and health risks for several elements, indicating that agricultural rural environments should not be regarded as low-exposure background areas during the haze season. Notably, lower PM_2.5_ mass concentrations did not necessarily correspond to lower health risks. Although PM_2.5_ concentrations at MC were lower than those at CMU, the estimated health risks were higher because of differences in elemental composition. This finding suggests that PM_2.5_ mass concentration alone may underestimate potential health impacts when highly toxic elements constitute a substantial proportion of particulate matter.

These findings have direct policy implications. Thailand’s haze response is coordinated through a single command center across nine northern provinces and a national action plan for PM_2.5_ that applies different urban, agricultural, and transboundary measures. Our finding that the roadside rural site (CT) had the highest health risk—even exceeding that of the agricultural site (MC)—suggests that current controls, which focus mainly on agricultural burning, may not adequately address traffic-influenced rural corridors. Additionally, although Thailand phased out leaded gasoline by 1996 [55], persistent Pb enrichment likely reflects non-exhaust traffic sources (brake wear and road dust), pointing to a regulatory gap beyond fuel-related controls. Therefore, effective haze mitigation strategies in northern Thailand should address not only regional biomass burning but also local emission sources, including traffic-related emissions, roadside dust resuspension, and agricultural biomass burning, particularly along transportation corridors such as CT. Furthermore, the present assessment considered only inhalation exposure to PM_2.5_-bound elements. The actual health burden experienced by local populations may also be influenced by other environmental and individual factors, including indoor radon exposure, airborne microbial communities (microbiome), occupational exposure, and health-related behaviors [86,87,88,89,90]. Potential interactions among these multiple exposures may result in additive or synergistic health effects and warrant further investigation to provide a more comprehensive assessment of health risks associated with haze episodes.

## 4. Conclusions

This study demonstrates that haze-season PM_2.5_ in Chiang Mai Province exhibits distinct spatial variation in both mass concentration and chemical composition across different land use settings. The roadside rural site (CT) consistently showed the highest PM_2.5_ concentrations, the greatest enrichment of several toxic trace metals, and the highest non-carcinogenic and carcinogenic risks, indicating that roadside rural communities may experience the greatest inhalation exposure during severe haze episodes. Although most metals showed low background contamination, Se, Cd, As, and Cr were identified as the principal contributors to anthropogenic enrichment and health risk. Qualitative source identification using PCA revealed covariance patterns among metals consistent with multiple overlapping sources, primarily biomass burning, traffic-related emissions, agricultural activities, and crustal or road dust; however, PCA does not quantify the relative contribution of each source, and receptor modeling approaches (e.g., PMF) would be required for quantitative source apportionment. These findings highlight the importance of considering PM_2.5_ chemical composition, in addition to particle mass concentration, when assessing health impacts during haze episodes. Accordingly, air quality management in northern Thailand should address both regional biomass-burning emissions and local emission sources, particularly traffic-related emissions and roadside dust resuspension in transportation corridors. Targeted mitigation strategies and public health interventions are needed to reduce population exposure, especially in roadside and rural communities affected by seasonal haze.

## Figures and Tables

**Table 1:** Concentration of PM_2.5_ and PM_2.5_-bound metals in Chiang Mai Province.

	Concentration of PM_2.5_ (µg m^−3^) and PM_2.5_-Bound Metals (ng m^−3^)						
	N	Mean	SD	Median	IQR	Min	Max
PM_2.5_	101	60.65	44.24	52.43	52.11	1.28	295.99
Al	101	2948.35	4284.71	1845.57	1575.22	693.08	38,522.73
As	96	19.87	21.21	10.61	26.98	1.28	107.95
Ba	101	13.32	11.89	11.89	16.26	0.81	53.41
Cd	56	1.80	1.42	1.47	2.46	0.41	5.31
Cr	100	38.71	47.83	25.07	23.14	6.71	414.77
Cu	70	6.44	5.19	6.12	6.98	1.09	28.19
Fe	101	335.58	585.28	221.17	214.35	42.53	5777.20
K	101	418.98	670.11	176.37	320.35	24.07	4909.64
Mn	101	12.39	9.03	9.28	8.45	2.42	46.15
Ni	35	9.88	5.50	8.22	3.91	2.65	31.67
Pb	95	11.73	15.38	7.86	8.68	1.87	125.00
Se	94	21.66	26.07	14.47	25.52	3.15	204.55
Zn	100	44.72	38.99	30.23	49.54	5.42	181.57

**Table 2:** Comparison of PM_2.5_ and PM_2.5_-bound metal concentrations in Chiang Mai Province and other studies.

Location	PM_2.5_ (µg m^−3^)	Al	As	Ba	Cd	Cr	Cu	Fe	K	Mn	Ni	Pb	Se	Zn	Ref.
WHO guideline	15.00		6.60		5.00	0.25				150.0	25.0	500.0			[12,13]
The initial Eastern Economic Corridor (EEC), Rayong, Thailand	21.80		2.87		1.01	8.69	8.06	393.0	541	31.6	1.2	37.7		476.0	[38]
Megacity with industry activities, Chengdu, China	85.1	195	10.70		2.25	3.29				29.2	1.8	40.7	3.1		[42]
Industry area with houses power plants, Dangjin, Republic of Korea	22.65	374.1	4.02	6.67		2.55	16.8	182.4		16.9		21.1		63.8	[53]
Industrial complex area, Yeosu-Gwangyang, Republic of Korea	17.45	355.2	2.71	5.76		2.41	3.9	181.5		12.1		9.4		44.0	[53]
Urban areas, Ahvaz, Iran	149.5	493	51		118	48.5	198	536		120	128	266		302	[54]
Industrial areas, Ahvaz, Iran	149.5	485	67		124	54	212	612		119	129	351		332	[54]
University area, Khon Kaen, Thailand	32.78	166.8			11.1		33.8	4.7		284.7		75.4		1.2	[51]
Residential area, Khon Kaen, Thailand	50.25	338.1			19.5		57.3	5.9		427.9		209.9		0.6	[51]
industrial zone, Khon Kaen, Thailand	44.48	1748			101.1		185.7	4.5		2296		272.9		4.4	[51]
Agricultural zone, Khon Kaen, Thailand	29.53	176			3.5		103.6	4		247.7		23.9		10.5	[51]
Urban area, Fuxin, China	39.68		22.5	20.5	1.9	9.5	14			18.7	6.1	66.4		294.7	[58]
Suburban area, Pathum Thani, Thailand	28.2					52.31	2.95	220.1	100.2	10.7	5.47			15.5	[52]
Commercial and residential areas, Jinan, China	41.7	403.7		6.7	1.3	12.3	10	1150		52.4	5.2	14.1	177.4	145.4	[45]
All (Chiang Mai)	60.65	2948	19.87	13.32	1.80	38.71	6.44	335.6	419.0	12.4	9.9	11.7	21.7	44.7	This study
CMU (urban area, university area)	62.46	1240	13.77	10.92	1.22	17.59	4.56	340.1	322.1	15.7	7.1	8.0	12.3	37.2	This study
MC (rural agricultural area)	45.74	2797	18.25	13.90	1.83	35.44	6.47	404.7	271.4	11.7	11.6	9.9	19.2	42.1	This study
CT (rural main road (mining road))	72.70	4081	25.18	14.23	2.46	54.31	8.20	272.3	605.2	11.1	10.4	15.4	29.9	51.6	This study

**Table 3:** Principal component analysis (PCA) and potential source identification of PM_2.5_-bound metals in Chiang Mai Province.

Metal	Component		
	1	2	3
Al	0.359	0.702	0.143
As	0.575	0.644	0.026
Ba	0.467	0.807	0.140
Cd	0.315	0.658	−0.210
Cr	−0.128	0.849	0.202
Cu	0.751	0.529	0.298
Fe	0.412	0.588	0.474
K	0.842	0.192	0.057
Mn	0.608	0.342	0.629
Ni	0.003	−0.008	0.895
Pb	0.922	0.108	0.033
Se	0.529	0.675	0.060
Zn	0.728	0.460	0.133
Eigenvalues	7.488	1.321	1.289
% of Variance	57.602	10.161	9.918
Cumulative %	57.602	67.763	77.681
Source Interpretation	Haze-related combustion mixed with vehicular contributions	Local dust resuspension and deposited combustion residues	Fuel combustion, mechanical abrasion, and soil dust

**Table 4:** Hazard quotient (HQ), hazard index (HI), inhalation cancer risk (CR), and total cancer risk (TCR) values for non-carcinogenic risks of PM_2.5_-bound metals in Chiang Mai Province.

Metal	All		CMU		MC		CT	
	HQ	CR	HQ	CR	HQ	CR	HQ	CR
Al	2.71 × 10^−1^		1.14 × 10^−1^		2.58 × 10^−1^		3.76 × 10^−1^	
As	6.10 × 10^−1^	1.35 × 10^−8^	4.22 × 10^−1^	9.34 × 10^−9^	5.60 × 10^−1^	1.24 × 10^−8^	7.73 × 10^−1^	1.71 × 10^−8^
Ba	1.23 × 10^−2^		1.00 × 10^−2^		1.28 × 10^−2^		1.31 × 10^−2^	
Cd	8.30 × 10^−2^	2.01 × 10^−6^	5.63 × 10^−2^	1.36 × 10^−6^	8.43 × 10^−2^	2.04 × 10^−6^	1.13 × 10^−1^	2.73 × 10^−6^
Cr	5.94 × 10^−1^	2.57 × 10^−4^	2.70 × 10^−1^	1.17 × 10^−4^	5.44 × 10^−1^	2.35 × 10^−4^	8.33 × 10^−1^	3.60 × 10^−4^
Cu	7.37 × 10^−5^		5.22 × 10^−5^		7.40 × 10^−5^		9.39 × 10^−5^	
Fe	2.21 × 10^−4^						1.79 × 10^−4^	
K								
Mn	1.14 × 10^−1^		1.45 × 10^−1^		1.07 × 10^−1^		1.02 × 10^−1^	
Ni	4.55 × 10^−1^	4.05 × 10^−10^	3.24 × 10^−1^	2.89 × 10^−10^	5.34 × 10^−1^	4.76 × 10^−10^	4.77 × 10^−1^	4.25 × 10^−10^
Pb	1.53 × 10^−3^	2.22 × 10^−11^			1.29 × 10^−3^	1.87 × 10^−11^	2.01 × 10^−3^	2.91 × 10^−11^
Se	4.99 × 10^−4^		2.82 × 10^−4^		4.43 × 10^−4^		6.89 × 10^−4^	
Zn	6.86 × 10^−5^		5.71 × 10^−5^		6.45 × 10^−5^		7.92 × 10^−5^	
HI or TCR	2.14	2.59 × 10^−4^	1.34	1.18 × 10^−4^	2.10	2.37 × 10^−4^	2.69	3.63 × 10^−4^

## Data Availability

The data that support the findings of this study are available from the corresponding author on reasonable request.

## Supplementary Materials

The following supporting information can be downloaded at: https://www.mdpi.com/article/10.3390/toxics14100869/s1, Table S1. Quality control values of metal measurement; Table S2. Background (reference) values of elements used for enrichment factor (EF) and geoaccumulation index (Igeo) calculations; Table S3. Literature-based source category tracers for PM_2.5_-bound metals used to interpret principal component analysis (PCA) results; Table S4. The values of parameters for non-carcinogenic and carcinogenic risk assessment; Table S5. The reference values for non-carcinogenic and carcinogenic risk assessment; Table S6. Comparisons of PM_2.5_ and PM_2.5_-bound metal concentrations in three different land use areas in Chiang Mai Province (15 January to 22 April 2020); Table S7. Pairwise comparisons of PM_2.5_ and metal concentrations among sampling sites using the Mann–Whitney U test (*p*-values); Table S8. Enrichment Factor (EF) values of PM_2.5_-bound metals in Chiang Mai Province; Table S9. Geoaccumulation index (Igeo) values of PM_2.5_-bound metals in Chiang Mai Province. References [91,92,93,94] are cited in Supplementary Materials.
