# Supplementary material for: Health Risk Assessment of Ambient PM2.5-Bound Metals During Haze Seasons in Urban and Rural Chiang Mai, Thailand
Source: Toxics. 2026 Sep 30;14(10):869. doi: 10.3390/toxics14100869 (PMC13709622; doi:10.3390/toxics14100869)
Supplement: Supplementary file 1 [file toxics-14-00869-s001.zip › toxics-4517108-supplementary.pdf]

Supplementary Information

Health Risk Assessment of Ambient $PM_{2.5}$-Bound Metals During Haze Seasons in Urban and Rural Chiang Mai, Thailand

Kawinwut Somsunun[1,2,†], Teetawat Santijitpakdee [1,3,†], Turenjai Doolgindachbaporn[4], Vanisa Surapipith[5], Sawaeng Kawichai[1,*] and Tippawan Prapamontol[1,*]

1 Research Institute for Health Sciences (RIHES), Chiang Mai University, Chiang Mai 50200, Thailand; kawinwut.som@cmu.ac.th (K.S.); santijitpakdee.t@gmail.com (T.S.)
2 Office of Research Administration, Chiang Mai University, Chiang Mai 50200, Thailand
3 School of Health Sciences Research (SHSR), Research Institute for Health Sciences (RIHES), Chiang Mai University, Chiang Mai 50200, Thailand
4 Department of Environmental Science, Faculty of Science, Khon Kaen University, Khon Kaen 40002, Thailand; turdoo@gmail.com
5 Regional Resource Centre for Asia and the Pacific at Asian Institute of Technology (AIT RRC.AP),
Pathum Thani 12120, Thailand; vanisa.surapipith@rrcap.ait.ac.th
* Correspondence: sawaeng.kaw@cmu.ac.th (S.K.); tippawan.prapamontol@cmu.ac.th (T.P.)
† These authors contributed equally to this work.

Table S1 Quality control values of metal measurement

Table S2 Background (reference) values of elements used for enrichment factor (EF) and geoaccumulation index (Igeo) calculations.

Table S3. Literature-based source category tracers for $PM_{2.5}$-bound metals used to interpret principal component analysis (PCA) results

Table S4. The values of parameters for non-carcinogenic and carcinogenic risk assessment

Table S5. The reference values for non-carcinogenic and carcinogenic risk assessment

Table S6 Comparisons of $PM_{2.5}$ and $PM_{2.5}$-bound metal concentrations in three different land-use areas in Chiang Mai Province (15th January to 22nd April 2020)

Table S7 Pairwise comparisons of $PM_{2.5}$ and metal concentrations among sampling sites using the Mann-Whitney U test (P-values).

Table S8 Enrichment Factor (EF) values of $PM_{2.5}$-bound metals in Chiang Mai Province

Table S9 Geoaccumulation Index (Igeo) values of $PM_{2.5}$-bound metals in Chiang Mai Province

Table S1 Quality control values of metal measurement

Elements	% Detection	LOD (ng m-3)	Standard recovery ± SD (%)	SRM 1648a NIST			% R.S.D	Linearity r^2
				SRM references (%, mg kg-1)	Measured (mg kg-1)	% SRM Recovery		
Al	100%	0.00	80.0 ± 0.0	3.43%	2.69%	78.5	8.96	0.9999
As	95%	2.57	79.0 ± 9.3	115.5	93.1	80.6	1.94	0.9999
Ba	100%	1.62	96.4 ± 7.2	N/A	N/A	N/A	2.04	0.9994
Cd	55%	0.82	91.3 ± 6.3	73.7	61.4	83.3	2.26	0.9994
Cr	99%	1.89	95.0 ± 12.3	402	282	70.3	5.63	0.9998
Cu	69%	2.19	98.0 ± 7.4	610	541	88.7	2.08	0.9999
Fe	100%	42.34	87.5 ± 14.1	3.92%	2.38%	60.8	3.24	0.9997
K	100%	48.15	94.5 ± 68.9	1.056%	0.66%	62.9	6.09	0.9999
Mn	100%	1.43	93.3 ± 6.3	790	612	77.5	2.06	0.9996
Ni	35%	1.40	93.6 ± 6.2	81.1	59.8	73.7	2.41	0.9998
Pb	94%	3.75	88.0 ± 7.4	0.655%	0.510%	78.4	1.80	0.9998
Se	93%	6.29	67.7 ± 8.3	N/A	N/A	N/A	2.26	0.9999
Zn	99%	2.99	84.5 ± 8.5	4,800	3,465	72.2	2.11	0.9995

Table S2 Background (reference) values of elements used for enrichment factor (EF) and geoaccumulation index (Igeo) calculations.

**Elements**	**Background (reference) values (ppm)**	**Referrence**
Al	80974.14	Kheamsiri K et al.,2026 [1]
As	4.37	Kheamsiri K et al.,2026 [1]
Ba	2627.94	Kheamsiri K et al.,2026 [1]
Cd	0.10	McLennan, 2001 [2]
Cr	14.57	Kheamsiri K et al.,2026 [1]
Cu	18.58	Kheamsiri K et al.,2026 [1]
Fe	29375.30	Kheamsiri K et al.,2026 [1]
K	56450.29	Kheamsiri K et al.,2026 [1]
Mn	1161.69	Kheamsiri K et al.,2026 [1]
Ni	11.07	Kheamsiri K et al.,2026 [1]
Pb	31.97	Kheamsiri K et al.,2026 [1]
Se	0.05	McLennan, 2001 [2]
Zn	106.11	Kheamsiri K et al.,2026 [1]

Table S3. Literature-based source category tracers for $PM_{2.5}$-bound metals used to interpret principal component analysis (PCA) results

Elements	Source Category Tracer	Reference
K	Biomass burning	[3-7]
Pb, Cu, Zn	Traffic/combustion emissions	[8-16]
Al, Ba, Fe	Crustal/mineral dust	[8,17-20]
Cr, Cd, As, Se	Anthropogenic (traffic + agricultural + combustion)	[8,17,21-27]
Ni, Mn	Fuel combustion/mechanical abrasion	[8,10,22,24,28-30]

Table S4. The values of parameters for non-carcinogenic and carcinogenic risk assessment

Abbrev	Parameter	Value	Unit	References
Non-carcinogenic risk				
AT_n	Averaging time	210,240	days	Huang W et al., Kawichai S et al. 2022 [31,32]
ED	Exposure duration	24	years	US EPA[33]
EF	Exposure frequency	350	days $year^{-1}$	US EPA[33]
ET	Exposure time	24	hours day^{-1}	US EPA[33]
E_i	The deposition fraction of $PM_{2.5}$ that can pass though lungs	0.48	mg day^{-1}	Kawichai S et al. 2022, Yang et al. 2022[32,34]
Carcinogenic Risk				
AT_n	Averaging time	613,200	days	Huang W et al. 2025, Kawichai S et al. 2022[31,32]
ED	Exposure duration	70	years	US EPA[33]
EF	Exposure frequency	350	days $year^{-1}$	US EPA[33]
ET	Exposure time	24	hours day^{-1}	US EPA[33]
E_i	The deposition fraction of $PM_{2.5}$ that can pass though lungs	0.48	mg day^{-1}	Kawichai S et al. 2022, Yang et al. 2022 [32,34]

Table S5. The reference values for non-carcinogenic and carcinogenic risk assessment

Elements	Non-carcinogenic	Carcinogenic
	RfC_{inh}	IUR_x
Al	5.00×10^{-3} [a]	
As	1.50×10^{-5} [a]	4.30×10^{-3} [a]
Ba	5.00×10^{-4} [a]	
Cd	1.00×10^{-5} [a]	7.05 [d]
Cr (VI)	3.00×10^{-5} [a]	42 [d]
Cu	4.02×10^{-2} [c]	
Fe	7.00×10^{-1} [a]	
Mn	5.00×10^{-5} [a]	
Ni	1.00×10^{-5} [a]	2.60×10^{-4} [a]
Pb	3.52×10^{-3} [c]	1.20×10^{-5} [f]
Se	2.00×10^{-2} [a]	
Zn	3.00×10^{-1} [a]	

a: US EPA (RSL)[33]

b: Ferreira-Baptista and De Miguel., 2005[35]

c: US EPA 2011

c: Doyi et al., 2019[36]

d: Wignall et al., 2018[37]

e: OEHHA[38]

Table S6 Comparisons of $PM_{2.5}$ and $PM_{2.5}$-bound metal concentrations in three different land-use areas in Chiang Mai Province (15th January to 22nd April 2020)

Parameters	Concentration of $PM_{2.5}$ (μg m^{-3}) and $PM_{2.5}$-bound metals (ng m^{-3})						
	CMU (n=24)		MC (n=36)		CT (n=41)		P-value
	Median	IQR	Median	IQR	Median	IQR	
$PM_{2.5}$	50.35	55.08	42.10	27.87	64.90	36.10	0.007
Al	1058.32	556.73	1981.20	1480.05	2287.12	2060.03	< 0.001
As	5.37	23.16	7.57	28.52	12.35	35.30	0.064
Ba	12.11	6.68	11.74	18.63	8.40	22.77	0.754
Cd	0.67	1.09	2.16	2.50	2.28	3.27	0.032
Cr	13.53	17.37	25.53	20.06	31.50	44.67	< 0.001
Cu	3.43	4.34	6.47	7.65	7.34	10.29	0.222
Fe	216.67	437.43	232.32	146.19	211.94	185.06	0.929
K	184.56	463.72	180.72	241.15	162.52	440.65	0.577
Mn	9.51	19.53	9.10	5.72	8.18	7.70	0.288
Ni	7.10	1.41	10.27	7.66	9.49	7.56	0.060
Pb	7.15	6.46	9.33	5.45	9.92	13.36	0.330
Se	7.70	20.45	12.58	26.06	17.57	26.06	0.010
Zn	29.28	39.63	31.01	34.97	26.92	63.78	0.576

Table S7 Pairwise comparisons of $PM_{2.5}$ and metal concentrations among sampling sites using the Mann-Whitney U test (P-values).

Parameters	CMU vs. MC	CMU vs. CT	MC vs. CT
$PM_{2.5}$	0.546	0.109	**0.001**
Al	**< 0.001**	**< 0.001**	0.312
As	0.461	**0.030**	0.095
Ba	0.700	0.838	0.411
Cd	0.094	**0.015**	0.204
Cr	**< 0.001**	**< 0.001**	0.126
Cu	0.205	0.106	0.592
Fe	0.916	0.967	0.639
K	0.571	0.654	0.312
Mn	0.245	0.133	0.585
Ni	0.055	**0.025**	0.957
Pb	0.225	0.188	0.562
Se	0.175	**0.003**	0.067
Zn	0.350	0.346	0.967

Notes: Values presented in the table are P-values calculated using the Mann-Whitney U test. Bold values indicate statistically significant differences (P-value< 0.05).
Abbreviations: CMU = Chiang Mai University site; MC = Mae Chaem District; CT = Chom Thong District.

Table S8 Enrichment Factor (EF) values of $PM_{2.5}$-bound metals in Chiang Mai Province

EF	All		CMU		MC		CT		P-value
	Median	IQR	Median	IQR	Median	IQR	Median	IQR	
Al	1	-	1	-	1	-	1	-	-
As	80.06	245.79	61.21	503.44	47.17	334.52	86.41	152.53	0.262
Ba	0.14	0.26	0.28	0.26	0.13	0.32	0.07	0.17	< 0.001
Cd	527.99	1,130.7	382.49	1,107.63	935.22	1196.2	534.18	1,108.09	0.695
Cr	70.67	38.29	60.88	90.89	70.56	30.35	73.35	31.47	0.683
Cu	10.69	20.57	10.37	26.84	17.08	21.87	8.27	20.72	0.138
Fe	0.25	0.2	0.73	0.97	0.28	0.15	0.21	0.12	< 0.001
K	0.12	0.25	0.19	0.73	0.11	0.21	0.11	0.17	0.034
Mn	0.28	0.46	0.85	0.79	0.29	0.22	0.22	0.13	< 0.001
Ni	48.31	36.07	54.2	10.18	47.37	34.02	29.44	44.14	0.072
Pb	8.56	11.45	14.13	18.34	8.11	10.63	8.28	11.81	0.059
Se	9,917.76	18,405.63	6,723.67	32,099.61	9,682.02	26,625.99	10,572.99	10,986.9	0.345
Zn	10.38	19.71	14.84	35.03	10.95	21.3	8.54	14.79	0.089

Table S9 Geoaccumulation Index (Igeo) values of $PM_{2.5}$-bound metals in Chiang Mai Province

Igeo	All		CMU		MC		CT		P-value
	Median	IQR	Median	IQR	Median	IQR	Median	IQR	
Al	-1.82	0.34	-2.06	0.21	-1.79	0.28	-1.73	0.34	< 0.001
As	0.21	0.89	-0.09	0.99	0.06	0.98	0.27	0.73	0.092
Ba	-2.51	0.87	-2.51	0.26	-2.49	0.86	-2.67	1.11	0.704
Cd	1.00	0.85	0.62	0.56	1.17	0.85	1.19	0.81	0.032
Cr	0.06	0.36	-0.21	0.51	0.07	0.29	0.16	0.47	< 0.001
Cu	-0.66	0.58	-0.91	0.45	-0.65	0.7	-0.58	1.02	0.246
Fe	-2.3	0.41	-2.31	0.77	-2.28	0.28	-2.32	0.36	0.929
K	-2.67	0.62	-2.66	0.7	-2.67	0.58	-2.68	0.73	0.537
Mn	-2.27	0.36	-2.26	0.59	-2.28	0.27	-2.33	0.37	0.29
Ni	-0.31	0.2	-0.37	0.09	-0.21	0.34	-0.24	0.31	0.06
Pb	-0.79	0.46	-0.83	0.41	-0.71	0.3	-0.68	0.62	0.327
Se	2.24	0.97	2.01	0.94	2.31	0.99	2.31	0.52	0.235
Zn	-0.72	0.61	-0.74	0.6	-0.71	0.46	-0.77	0.72	0.576

References

1 Kheamsiri, K.; Akata, N.; Kranrod, C.; Tazoe, H.; Thumvijit, T.; Rosianna, I.; Kuwata, H.; Khetanun, K.; Yimyam, N.; Unno, Y.; et al. Geochemical and Radiological Characterization of Granitic-Derived Highland Coffee Soils in Chiang Mai, Thailand. Geosciences 2026, 16, 110, doi:10.3390/geosciences16030110.

2 McLennan, S. Relationships between the trace element composition of sedimentary rocks and upper continental crust. Geochemistry Geophysics Geosystems - GEOCHEM GEOPHYS GEOSYST 2001, 2, doi:10.1029/2000GC000109.

3 Saksakulkrai, S.; Chantara, S.; Kraisitnitikul, P.; Srivastava, D.; Shi, Z. Unveiling the origins of Northern Thailand’s haze: comprehensive chemical characterization and source apportionment of $PM_{2.5}$ using targeted molecular markers. Journal of Environmental Sciences 2026, 159, 635-648, doi:https://doi.org/10.1016/j.jes.2025.04.040.

4 Kraisitnitikul, P.; Thepnuan, D.; Chansuebsri, S.; Yabueng, N.; Wiriya, W.; Saksakulkrai, S.; Shi, Z.; Chantara, S. Contrasting compositions of $PM_{2.5}$ in Northern Thailand during La Niña (2017) and El Niño (2019) years. Journal of Environmental Sciences 2024, 135, 585-599, doi:10.1016/j.jes.2022.09.026.

5 Han, Y.-S.; Eun, D.-M.; Lee, G.; Gong, S.Y.; Youn, J.-S. Enhancement of $PM_{2.5}$ source appointment in a large industrial city of Korea by applying the elemental carbon tracer method for positive matrix factorization (PMF) model. Atmospheric Pollution Research 2023, 14, 101910, doi:https://doi.org/10.1016/j.apr.2023.101910.

6 Sharma, S.K.; Mandal, T.K. Elemental Composition and Sources of Fine Particulate Matter ($PM_{2.5}$) in Delhi, India. Bulletin of Environmental Contamination and Toxicology 2023, 110, 60, doi:10.1007/s00128-023-03707-7.

7 Xu, J.; Jia, C.; Yu, H.; Xu, H.; Ji, D.; Wang, C.; Xiao, H.; He, J. Characteristics, sources, and health risks of $PM_{2.5}$-bound trace elements in representative areas of Northern Zhejiang Province, China. Chemosphere 2021, 272, 129632, doi:https://doi.org/10.1016/j.chemosphere.2021.129632.

8 Wang, J.; Zhang, S.; Qiu, X.; Li, K.; Li, J.; Ren, Y.; Zhu, C.; Zhang, X. Characteristics and Health Risks of $PM_{2.5}$-bound Metals in a Central City of Northern China: A One-year Observation Study. Aerosol and Air Quality Research 2024, 24, 230165, doi:10.4209/aaqr.230165.

9 Kayee, J.; Sompongchaiyakul, P.; Sanwlani, N.; Bureekul, S.; Wang, X.; Das, R. Metal Concentrations and Source Apportionment of $PM_{2.5}$ in Chiang Rai and Bangkok, Thailand during a Biomass Burning Season. ACS Earth and Space Chemistry 2020, 4, 1213-1226, doi:10.1021/acsearthspacechem.0c00140.

10 Molnár, P.; Gustafson, P.; Johannesson, S.; Boman, J.; Barregård, L.; Sällsten, G. Domestic wood burning and $PM_{2.5}$ trace elements: Personal exposures, indoor and outdoor levels. Atmospheric Environment 2005, 39, 2643-2653, doi:https://doi.org/10.1016/j.atmosenv.2005.01.016.

11 Zhao, X.; Shen, Z.; Han, F.; Bharti, B.; Feng, S.; Du, J.; Li, Y. Pollution characteristics and health risk assessment of heavy metals in $PM_{2.5}$ in Fuxin, China. Environmental Geochemistry and Health 2024, 46, 511, doi:10.1007/s10653-024-02275-x.

12 Wang, X.; Gronstal, S.; Lopez, B.; Jung, H.; Chen, L.W.A.; Wu, G.; Ho, S.S.H.; Chow, J.C.; Watson, J.G.; Yao, Q.; et al. Evidence of non-tailpipe emission contributions to $PM_{2.5}$ and PM10 near southern California highways. Environmental Pollution 2023, 317, 120691, doi:https://doi.org/10.1016/j.envpol.2022.120691.

13 Hsu, C.Y.; Chi, K.H.; Wu, C.D.; Lin, S.L.; Hsu, W.C.; Tseng, C.C.; Chen, M.J.; Chen, Y.C. Integrated analysis of source-specific risks for PM(2.5)-bound metals in urban, suburban, rural, and industrial areas. Environ Pollut 2021, 275, 116652, doi:10.1016/j.envpol.2021.116652.

14 Niu, Y.; Wang, F.; Liu, S.; Zhang, W. Source analysis of heavy metal elements of $PM_{2.5}$ in canteen in a university in winter. Atmospheric Environment 2021, 244, 117879, doi:https://doi.org/10.1016/j.atmosenv.2020.117879.

15 Cui, L.; Wu, Z.; Han, P.; Taira, Y.; Wang, H.; Meng, Q.; Feng, Z.; Zhai, S.; Yu, J.; Zhu, W.; et al. Chemical content and source apportionment of 36 heavy metal analysis and health risk assessment in aerosol of Beijing. Environmental Science and Pollution Research 2020, 27, 7005-7014, doi:10.1007/s11356-019-06427-w.

16 Alves, D.D.; Riegel, R.P.; Klauck, C.R.; Ceratti, A.M.; Hansen, J.; Cansi, L.M.; Pozza, S.A.; de Quevedo, D.M.; Osório, D.M.M. Source apportionment of metallic elements in urban atmospheric particulate matter and assessment of its water-soluble fraction toxicity. Environmental Science and Pollution Research 2020, 27, 12202-12214, doi:10.1007/s11356-020-07791-8.

17 Huang, W.; Kuang, D.; Qin, F.; Luo, L.; Yao, J.; Song, S.; Sun, J.; Huang, J.; Wang, C.; Lu, R.; et al. Health risks and sources of PM(2.5)-bound heavy metals in Southwest China: evidence from a 9-year monitoring program. BMC Public Health 2025, 25, 3872, doi:10.1186/s12889-025-24596-x.

18 Wu, B.; Li, L.; Guo, S.; Li, Y. Source apportionment of heavy metals in the soil at the regional scale based on soil-forming processes. J Hazard Mater 2023, 448, 130910, doi:10.1016/j.jhazmat.2023.130910.

19 Jandacka, D.; Brna, M.; Durcanska, D.; Kovac, M. Characterization of Road Dust, PMx and Aerosol in a Shopping–Recreational Urban Area: Physicochemical Properties, Concentration, Distribution and Sources Estimation. Sustainability 2023, 15, 12674.

20 Guo, Q.; Li, L.; Zhao, X.; Yin, B.; Liu, Y.; Wang, X.; Yang, W.; Geng, C.; Wang, X.; Bai, Z. Source Apportionment and Health Risk Assessment of Metal Elements in $PM_{2.5}$ in Central Liaoning's Urban Agglomeration. Atmosphere 2021, 12, 667.

21 Kawichai, S.; Bootdee, S.; Chantara, S. Health risk assessments and source apportionment of $PM_{2.5}$-bound heavy metals in the initial eastern economic corridor (EEC): A case study of Rayong Province, Thailand. Atmospheric Pollution Research 2024, 15, 102205, doi:https://doi.org/10.1016/j.apr.2024.102205.

22 Luiza Fernandes Seares, A.; de Oliveira Souza, M.; Lionel-Mateus, V.; De Almeida, C.M.S. Optimization of operational ICP OES parameters and application to PM10 monitoring associated to sugarcane burning. Microchemical Journal 2021, 163, 105917, doi:https://doi.org/10.1016/j.microc.2020.105917.

23 Yu, Y.; Cao, J. Chemical Fingerprints and Source Profiles of PM10 and $PM_{2.5}$ from Agricultural Soil in a Typical Polluted Region of Northwest China. Aerosol and Air Quality Research 2023, 23, 220419, doi:10.4209/aaqr.220419.

24 Isley, C.F.; Taylor, M.P. Atmospheric remobilization of natural and anthropogenic contaminants during wildfires. Environmental Pollution 2020, 267, 115400, doi:https://doi.org/10.1016/j.envpol.2020.115400.

25 Pandey, B.; Agrawal, M.; Singh, S. Assessment of air pollution around coal mining area: Emphasizing on spatial distributions, seasonal variations and heavy metals, using cluster and principal component analysis. Atmospheric Pollution Research 2014, 5, 79-86, doi:https://doi.org/10.5094/APR.2014.010.

26 Gao, Q.; Zhu, S.; Zhou, K.; Zhai, J.; Chen, S.; Wang, Q.; Wang, S.; Han, J.; Lu, X.; Chen, H.; et al. High enrichment of heavy metals in fine particulate matter through dust aerosol generation. Atmos. Chem. Phys. 2023, 23, 13049-13060, doi:10.5194/acp-23-13049-2023.

27 Varrica, D.; Lo Medico, F.; Zuccolini, M.V.; Miola, M.; Alaimo, M.G. Geochemical baseline values determination and spatial distribution of trace elements in topsoils: An application in Sicily region (Italy). Science of The Total Environment 2024, 955, 176951, doi:https://doi.org/10.1016/j.scitotenv.2024.176951.

28 Liu, K.; Shang, Q.; Wan, C.; Song, P.; Ma, C.; Cao, L. Characteristics and Sources of Heavy Metals in $PM_{2.5}$ during a Typical Haze Episode in Rural and Urban Areas in Taiyuan, China. Atmosphere 2018, 9, 2.

29 Li, C.; Bai, L.; Qin, J.; Guo, Y.; Wang, H.; Xu, X. Study on metal elements in indoor particulate matter: a case study of rural residential environment in Northeast China. Environ Geochem Health 2023, 45, 4867-4881, doi:10.1007/s10653-023-01543-6.

30 Kajino, M.; Hagino, H.; Fujitani, Y.; Morikawa, T.; Fukui, T.; Onishi, K.; Okuda, T.; Kajikawa, T.; Igarashi, Y. Modeling Transition Metals in East Asia and Japan and Its Emission

31 Huang, W.; Kuang, D.; Qin, F.; Luo, L.; Yao, J.; Song, S.; Sun, J.; Huang, J.; Wang, C.; Lu, R.; et al. Health risks and sources of PM(2.5)-bound heavy metals in Southwest China: evidence from a 9-year monitoring program. BMC Public Health 2025, 25, 3872, doi:10.1186/s12889-025-24596-x.

32 Kawichai, S.; Bootdee, S.; Sillapapiromsuk, S.; Janta, R. Epidemiological Study on Health Risk Assessment of Exposure to $PM_{2.5}$-Bound Toxic Metals in the Industrial Metropolitan of Rayong, Thailand. Sustainability 2022, 14, 15368, doi:10.3390/su142215368.

33 EPA, U. US EPA Regional Screening Levels (RSL) Calculator. Available online: https://epa-prgs.ornl.gov/cgi-bin/chemicals/csl_search (accessed on 20 Dec 2022).

34 Yang, D.; Li, Z.; Yue, Z.; Liu, J.; Zhai, Z.; Li, Z.; Gao, M.; Hu, A.; Zhu, W.; Ding, N.; et al. Variations in Sources, Composition, and Exposure Risks of $PM_{2.5}$ in both Pre-Heating and Heating Seasons. Aerosol and Air Quality Research 2022, 22, 210333, doi:10.4209/aaqr.210333.

35 Ferreira-Baptista, L.; De Miguel, E. Geochemistry and risk assessment of street dust in Luanda, Angola: A tropical urban environment. Atmospheric Environment 2005, 39, 4501-4512, doi:https://doi.org/10.1016/j.atmosenv.2005.03.026.

36 Doyi, I.N.Y.; Isley, C.F.; Soltani, N.S.; Taylor, M.P. Human exposure and risk associated with trace element concentrations in indoor dust from Australian homes. Environment International 2019, 133, 105125, doi:https://doi.org/10.1016/j.envint.2019.105125.

37 Wignall, J.A.; Muratov, E.; Sedykh, A.; Guyton, K.Z.; Tropsha, A.; Rusyn, I.; Chiu, W.A. Conditional Toxicity Value (CTV) Predictor: An <i>In Silico</i> Approach for Generating Quantitative Risk Estimates for Chemicals. Environmental Health Perspectives 2018, 126, 057008, doi:doi:10.1289/EHP2998.

38 Office of Environmental Health Hazard Assessment (OEHHA). Technical Support Document for Cancer Potency Factors 2009. Available online: https://oehha.ca.gov/air/crnr/technical-support-document-cancer-potency-factors-2009 (accessed on 20 April 2023).
